# Ca^2+^ Binding Enhanced Mechanical Stability of an Archaeal Crystallin

**DOI:** 10.1371/journal.pone.0094513

**Published:** 2014-04-11

**Authors:** Venkatraman Ramanujam, Hema Chandra Kotamarthi, Sri Rama Koti Ainavarapu

**Affiliations:** Department of Chemical Sciences, Tata Institute of Fundamental Research, Colaba, Mumbai, India; Universidad de Granada, Spain

## Abstract

Structural topology plays an important role in protein mechanical stability. Proteins with β-sandwich topology consisting of Greek key structural motifs, for example, I27 of muscle titin and ^10^FNIII of fibronectin, are mechanically resistant as shown by single-molecule force spectroscopy (SMFS). In proteins with β-sandwich topology, if the terminal strands are directly connected by backbone H-bonding then this geometry can serve as a “mechanical clamp”. Proteins with this geometry are shown to have very high unfolding forces. Here, we set out to explore the mechanical properties of a protein, M-crystallin, which belongs to β-sandwich topology consisting of Greek key motifs but its overall structure lacks the “mechanical clamp” geometry at the termini. M-crystallin is a Ca^2+^ binding protein from *Methanosarcina acetivorans* that is evolutionarily related to the vertebrate eye lens β and γ-crystallins. We constructed an octamer of crystallin, (M-crystallin)_8_, and using SMFS, we show that M-crystallin unfolds in a two-state manner with an unfolding force ∼90 pN (at a pulling speed of 1000 nm/sec), which is much lower than that of I27. Our study highlights that the β-sandwich topology proteins with a different strand-connectivity than that of I27 and ^10^FNIII, as well as lacking “mechanical clamp” geometry, can be mechanically resistant. Furthermore, Ca^2+^ binding not only stabilizes M-crystallin by 11.4 kcal/mol but also increases its unfolding force by ∼35 pN at the same pulling speed. The differences in the mechanical properties of apo and holo M-crystallins are further characterized using pulling speed dependent measurements and they show that Ca^2+^ binding reduces the unfolding potential width from 0.55 nm to 0.38 nm. These results are explained using a simple two-state unfolding energy landscape.

## Introduction

**Figure 1:**
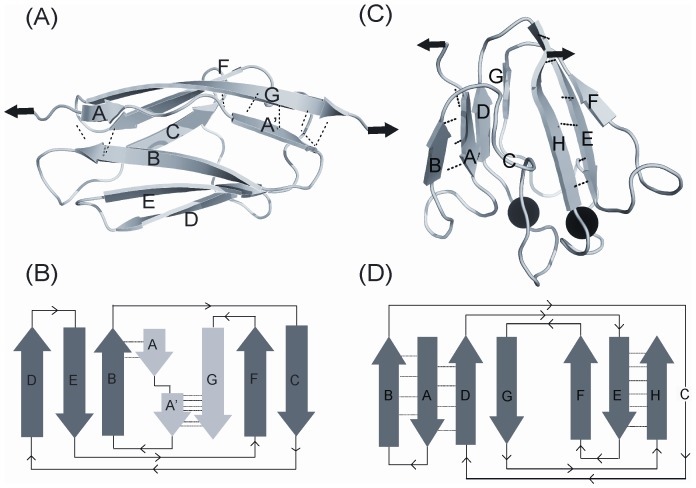
Structure and 2D topology diagram of two β-sandwich proteins with Greek key motifs used in this study. The pulling direction used in the single-molecule force spectroscopy (SMFS) experiments is shown by arrows. (*A*) NMR structure of I27 (PDB ID: 1TIT). Terminal β-strands A′ and G are directly connected by H-bonding, shearing this “mechanical-clamp” results in the mechanical unfolding of the protein. The rupture of H-bonds between A and B strands constitutes the less stable mechanical intermediate. (*B*) 2D topology diagram of I27. The five-stranded (BCDEF) ‘double’ Greek key (3,2)_3_ formed by overlapping (3,1)_N_ and (2,2)_C_ Greek keys (as defined by Hutchinson and Thornton [53]). (*C*) NMR structure of M-crystallin (PDB ID: 2K1W) bound to two Ca^2+^ ions (shown as black spheres). The terminal β-strands A and H are not directly bonded and they need to be “peeled” away from each other to unfold the protein. (*D*) 2D topology diagram of M-crystallin showing the two (3,1)_C_ Greek keys formed by ABCD and EFGH. In both cases, the backbone H-bonding around the terminal strands is shown.

Single-molecule force spectroscopy (SMFS) studies showed that the proteins with the classical β-sandwich topology consisting of Greek key motifs in their structure are generally mechanically resistant to unfolding [1]–[5]. Notable examples of this topology are I27 (the 27^th^ immunoglobulin-like domain from human cardiac titin), ^10^FNIII (the 10^th^ domain of type III from fibronectin), TNfn3 (the 3^rd^ fibronectin type III domain from human tenascin). In I27, the terminal A′ and G strands are directly connected through backbone H-bonding, which is often called a “mechanical clamp” geometry (Figure 1, *A* and *B*), whereas ^10^FNIII and TNfn3 lack this special feature. For this reason I27 unfolds at a higher force (∼200 pN) than ^10^FNIII and TNfn3, which unfold at lower forces (∼100 pN) and it was shown by experiments and simulations that the rupture of the H-bonds in the “mechanical clamp” of I27 leads to its mechanical unfolding [1], [5]–[8]. In a recent study, it was shown that two “mechanical clamps” in tandem make cohesins twice as strong as I27 and their reported unfolding forces are >400 pN [9]. Furthermore, the “mechanical clamp” geometry present in proteins with β-grasp topology is also attributed to their high mechanical stability [10], [11].

Because of their unparalleled mechanical strength, proteins with the “mechanical clamp” are often studied by SMFS. It has been recognized in recent studies that the interactions other than the “mechanical clamp” are also important in resistance to mechanical unfolding of proteins [5], [12], [13]. Hence, SMFS studies on a repertoire of proteins with diverse structures and topology are needed to fully understand the basis of mechanical resistance in proteins. There are different arrangements of β-strands in the β-sandwich topology proteins without the “mechanical clamp” geometry [14], [15]. These proteins with different β-strand arrangement (or connectivity) would provide a platform to test and expand our knowledge on the strand connectivity dependent mechanical stability. Therefore, it would be important to measure the mechanical properties of such topologies to further understand the relation between the structural topology and the protein mechanical stability. Here, we have chosen M-crystallin, a β-sandwich topology protein, with strand connectivity different from that of I27 and also without the “mechanical clamp”, to investigate its mechanical properties. The structures and the β-strand arrangements of I27 and M-crystallin are shown in Figure 1. Despite having β-sandwich topology, the β-strand arrangements in M-crystallin and I27 are quite different. In the case of I27, the terminal strands (A′ and G) are directly connected via backbone H-bonding. In M-crystallin, on the other hand, the terminal strands (A and H) are not directly connected but present in two β-sheets facing each other. M-crystallin is a Ca^2+^ binding protein from *Methanosarcina acetivorans*. Evolutionarily, it is related to the vertebrate eye lens β and γ-crystallin and its NMR structure has been shown to have two Ca^2+^ binding sites [16].

SMFS using atomic force microscope, when combined with polyprotein engineering, has provided valuable information on the mechanical properties of proteins for more than a decade. There have been excellent reviews on this topic highlighting the salient features of the technique [17]–[23]. In SMFS, tandem-linked proteins (polyproteins or chimeric proteins) are commonly used as they provide the fingerprints for identifying single-molecules. Pulling speed dependence on the mechanical unfolding processes provides the details of the unfolding energy landscape in terms of the potential width and the barrier of crossing from native state.

We report here the protein construction of octameric M-crystallin. Using fluorescence spectroscopy and circular dichroism, we show that the protein structure remains unperturbed upon polyprotein construction. Using isothermal titration calorimetry, we measure the dissociation constants for the two Ca^2+^ binding sites of M-crystallin in the polyprotein and we show that the ligand binding strength is also not altered. We demonstrate that M-crystallin by itself is mechanically stable and provides characteristic sawtooth patterns in single-molecule pulling experiments and its mechanical stability is further enhanced upon Ca^2+^ binding. We finally conclude that the Ca^2+^ binding enhanced mechanical stability is due to the stabilization of native state as well as the reduced unfolding potential width.

## Materials and Methods

### Protein engineering

The gene of M-crystallin was cloned into the pQE80L (Qiagen, Valencia, CA) expression vector using BamHI, BglII and KpnI restriction sites in a manner described by Carrion-Vazquez *et al* [1]. The gene of octameric M-crystallin was constructed by using iterative cloning and the corresponding protein was expressed and purified according to the procedure described Ramanujam *et al* [24]. For holo protein, 10 mM Tris buffer pH 7.5 containing 50 mM KCl and 10 mM CaCl_2_ was used. For experiments in apo conditions, the proteins were buffer exchanged with 10 mM Tris buffer, pH 7.5 containing 50 mM KCl, 2 mM EDTA and 2 mM EGTA followed by chelex treated 10 mM Tris buffer, pH 7.5 containing 50 mM KCl.

### Single-molecule force spectroscopy (SMFS)

Single-molecule pulling experiments were carried out on a custom-built atomic force microscope, whose details are described elsewhere [25]. Calibration of cantilevers was done using equipartition theorem before each pulling experiment [26]. In a typical pulling experiment ∼40 µl of (1 µM) protein in 10 mM Tris buffer (pH 7.5) with 50 mM KCl for apo protein and in 10 mM Tris buffer (pH 7.5) with 50 mM KCl and 10 mM CaCl_2_ for holo protein was kept on a gold-coated glass coverslip and incubated for 10 min. The cantilever was then calibrated in protein solution (spring constant ∼40 pN/nm) and further used for force extension (FX) experiments.

### Monte Carlo Simulations

Monte Carlo simulations were performed by pulling eight repeats of M-crystallin in tandem to obtain the unfolding energy landscape parameters as described previously by Rief et al [27]. A two-state energy landscape with a single barrier separating them was assumed. The tandem repeat M-crystallin was pulled at a constant speed and the force on the molecule was calculated using the following WLC model equation [28]. 
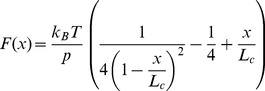
(1)where p and L_c_ denote the persistence length and contour length, respectively, and k_B_T is thermal energy at room temperature. As the tandem repeat is pulled, the probability of unfolding (P_u_) of any of the folded units is calculated using 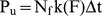, where N_f_ is the number of folded M-crystallin units, Δt is the time step, and 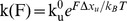. An unfolding event was registered when P_u_ is equal to a random number between 0 and 1. The unfolding event was followed by an increase in the contour length of the polypeptide and the number of folded units was decreased by one. The procedure was repeated till all the units in the tandem repeat get unfolded and the unfolding force histograms were constructed. The values of k_u_^0^ and Δx_u_ were varied such that the pulling speed dependent force histograms obtained from simulations are in agreement with the experimental data.

In addition, we have also extracted the unfolding energy landscape parameters using Bell-Evans-Ritchie model [28]–[31] as shown in the following expression.
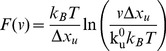
(2)where F(ν) is unfolding force, ν is loading rate, Δx_u_ is distance to the transition state, k_u_^0^ is rate constant for spontaneous unfolding. The loading rate ν was calculated by multiplying the pulling speed with the average spring constant of our cantilever (40 pN/nm).

### Estimating transition state energy barrier and spring constant of the unfolding potential

We followed the method suggested by Dietz *et al* [32], to calculate the transition state energy barrier and spring constant of the unfolding potential. The transition state barrier height (ΔG^‡^) was calculated using the Arrhenius equation 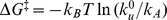, where k_B_ is Boltzmann's constant, T is temperature ( = 298K), k_u_^0^ is the rate constant for spontaneous unfolding and k_A_ is the Arrhenius frequency factor. Here, k_A_ is taken as 10^9^ s^−1^ [32], [33]. The unfolding potential is assumed to be harmonic and its spring constant (k_s_) is calculated using the equation 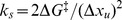, where Δx_u_ is the distance to the transition state or width of the unfolding potential. The ΔG and k_s_ are calculated from the k_u_^0^ and Δx_u_ values obtained in Monte Carlo simulations.

## Results

### Protein construction and structural analysis by circular dichroism (CD) and fluorescence spectroscopy

Polyprotein of M-crystallin containing eight domains, (M-crystallin)_8_, has been constructed. The molecular weights of monomer and octamer are 10.9 kDa and 87.2 kDa, respectively. The yield of the protein was estimated to be 6 mg/L for octamer and 30 mg/L for monomer. SDS PAGE analysis and size exclusion chromatography results are given in supporting material (Figure S1 in File S1). The secondary and tertiary structural elements between monomer and octamer were compared using circular dichroism spectroscopy (CD) (Figure S2 in File S1). In the far UV region (205–250 nm) CD spectra, the minimum ellipticity is at 220 nm signifying the β-sheet content in the protein structure (Figure S2 in File S1). The results of the monomer are identical to that of M-crystallin monomer reported earlier by Barnwal *et al* [16]. The CD spectra of holo protein were obtained in the presence of 10 mM CaCl_2_ (Figure S2 in File S1). There is a slight increase in the ellipticity upon Ca^2+^ binding. However, the CD spectra of monomer and octamer are identical, both in apo and holo conditions, indicating that the secondary structural characteristics remain the same in the polyprotein. Also, M-crystallin has aromatic amino acids (two tryptophans and six tyrosines). We have measured the CD spectra in the near-UV region (250–300 nm), where aromatic amino acids absorb and would give a non-zero CD if they are in asymmetric environment in the protein structure (Figure S2, *C* and *D* in File S1). In this wavelength region, spectra show a non-zero positive signal, indicating an asymmetric environment around the aromatic residues. This characteristic property is not altered upon Ca^2+^ binding (Figure S2, *C* and *D* in File S1). From the similarity in the near-UV region CD spectra of monomer and octamer it is evident that the asymmetric environment sensed by the aromatic residues is the same and hence the overall tertiary structure is preserved in polyprotein engineering. A previous study by Ramanujam *et al* [24] showed that the structural properties of monomer and dimer of M-crystallin are highly similar.

M-crystallin has two tryptophans at positions 46 and 59, and their fluorescence properties were measured for monomer and octamer in native and denatured conditions (Figure S3 in File S1). The samples were excited at 295 nm and the emission spectra were recorded in the range 310–400 nm. In the native conditions, the fluorescence maximum was at 332 nm and the spectra of M-crystallin monomer and octamer are identical. The spectra were recorded in the denaturing buffer containing 6 M GdnHCl and the emission maximum shifted to 352 nm for both monomer and octamer. As tryptophan fluorescence properties are known to be environment sensitive and from the experiments on monomer and octamer it is evident that the structural properties of M-crystallin octamer are unaltered from those of monomer, both in native and denaturing conditions.

### Ca^2+^ binding and M-crystallin stabilization

Although Ca^2+^ binding is indirectly indicated by the subtle changes in circular dichroic spectra, we have independently confirmed it using isothermal titration calorimetry (ITC) experiments. Figure S4 in File S1 shows the ITC thermograms of the monomer and the octamer. The ITC data could be best fitted with two-site sequential binding model for monomer as well as octamer. The resulting thermodynamic parameters are given in Table 1. M-crystallin has two Ca^2+^ binding sites as shown in Figure 1. These binding sites differ in their binding strengths and we obtain the dissociation constants K_d1_ ∼30 µM and K_d2_ ∼200 µM for site I and site II, respectively, in M-crystallin monomer, which are in complete agreement with those reported by Barnwal *et al* [16]. The K_d1_ and K_d2_ from our measurements on the octamer are ∼30 µM and ∼166 µM, respectively, and they are comparable to that of the monomer. Furthermore, the Gibbs free energies of Ca^2+^ binding for the two sites (ΔG_1_∼−6.2 kcal/mol and ΔG_2_∼−5.2 kcal/mol) also match with that of the monomer (see Table 1). This means that M-crystallin binds Ca^2+^ with moderate affinity and that the free energy of holo protein (ΔG_1_+ΔG_2_ = ΔG∼−11.4 kcal/mol) is substantially lower than that of the apo protein. Hence, the Ca^2+^ binding sites are not only well preserved in polyprotein construction but they have retained the same binding affinity and free energy of binding.

**Table 1:** Thermodynamic parameters of two Ca^2+^ binding sites in M-crystallin monomer and octamer.

Parameter	Monomer	Octamer
K_d1_ (µM)	31±7	31±6
ΔH_1_ (kcal/mol)	−7.7±0.1	−8.5±0.1
ΔS_1_ (cal/mol/K)	−5.2±0.8	−7.9±0.8
ΔG_1_ (kcal/mol)	−6.2±0.2	−6.2±0.2
K_d2_ (µM)	200±60	166±60
ΔH_1_ (kcal/mol)	−3.2±0.1	−3.2±0.1
ΔS_1_ (cal/mol/K)	6.2±1.0	6.6±1.1
ΔG_2_ (kcal/mol)	−5.0±0.2	−5.2±0.2

### Mechanical unfolding experiments on (M-crystallin)_8_

**Figure 2:**
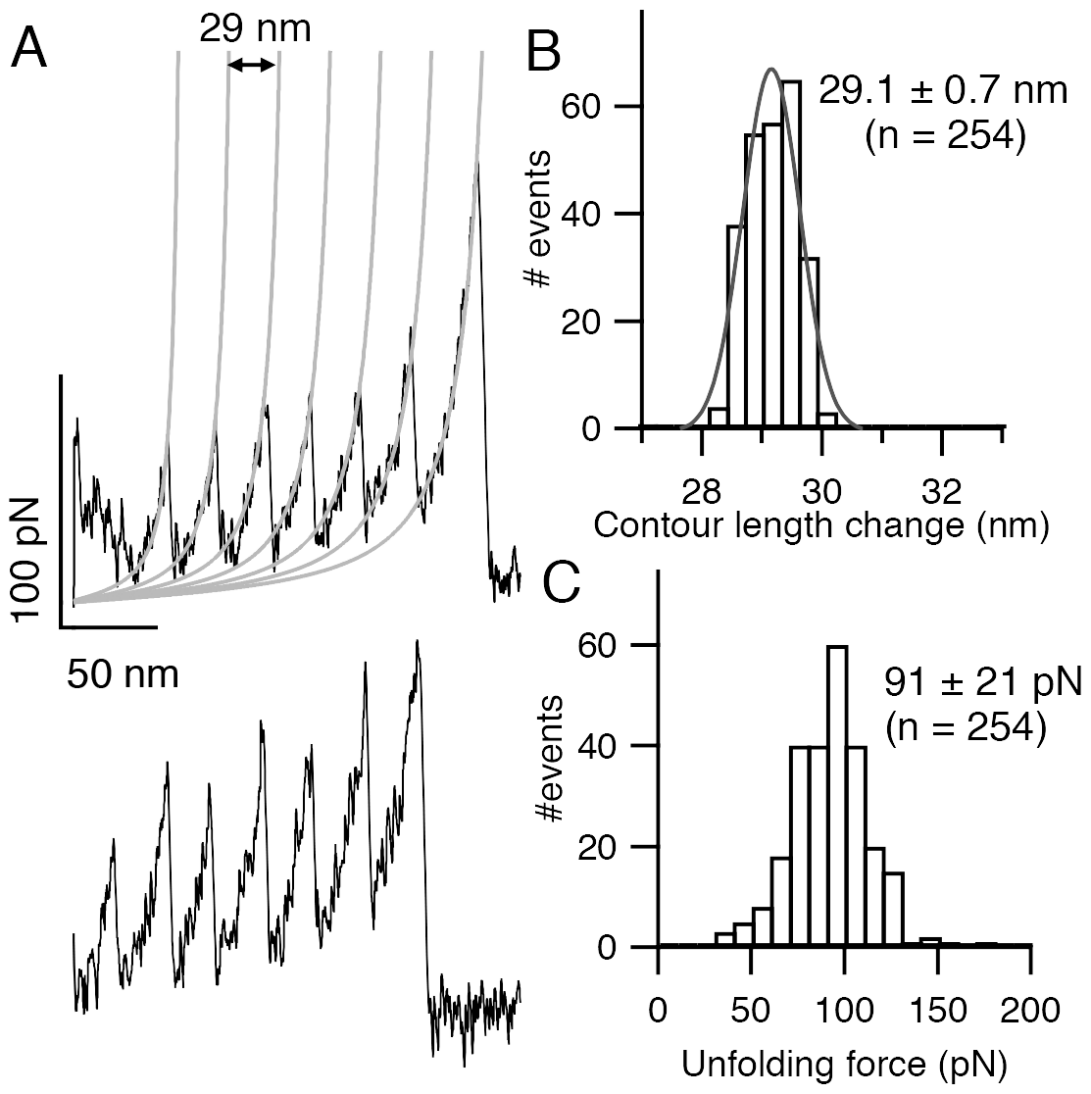
Mechanical unfolding of (M-crystallin)_8_ using SMFS. (*A*) A pair of typical single-molecule force extension (FX) traces of apo protein (black). A series of equidistant force peaks in FX traces indicating the sequential unfolding of individual M-crystallin units in the octamer during the mechanical stretching (pulling speed is 1000 nm/s). The unfolding force peaks in sawtooth pattern are fitted with WLC model (grey). The contour length change is ∼29 nm and the unfolding force is ∼90 pN. Histograms of contour length change fitted to Gaussian distribution (*B*) and unfolding force (*C*) are shown.

Once it is confirmed that M-crystallin units in octamer have similar structural and Ca^2+^ binding properties as that of the monomer, we set out to perform pulling experiments on (M-crystallin)_8_ using SMFS. Figure 2*A* shows two single-molecule mechanical unfolding traces of the octamer obtained at a pulling speed of 1000 nm/s. The force extension (FX) traces show a series of force peaks with unfolding forces ∼90 pN occurring with a regular interval resulting in a sawtooth-like pattern. The sawtooth pattern was fitted to a series of worm-like chain (WLC) model curves. WLC model describes the entropic elasticity of polymers when subjected to stretching forces and provides the contour length of the polymer [28]. M-crystallin consists of 85 aa in its structure. The expected contour length increment based on the size of the protein (85 aa×0.36 nm – N-C distance) is ∼29.1 nm. Here, the average N-C distance ∼1.5 nm is deduced from the NMR structure of M-crystallin (PDB ID: 2K1W). The contour length difference of the fitted WLC curves between a pair of adjacent force peaks is measured to be ∼29 nm, which is in accord with the expected value (Figure 2*A*). Hence, each force peak in the sawtooth pattern corresponds to the complete unfolding of one M-crystallin unit in the octamer. It also confirms that each M-crystallin unit unfolded in an all-or-none fashion without giving any discernible intermediate. The sawtooth pattern with ∼29 nm spaced force peaks is the fingerprint of M-crystallin unfolding. In further analysis, all the FX traces with at least four unfolding events were used to make histograms of contour length change and unfolding force. The histograms are shown in Figure 2 *B* and *C*. The measured value of unfolding force is 91±21 pN (average ± SD) and the contour length change is 29.1±0.4 nm from 254 unfolding events (Table 2). The unfolding force of M-crystallin is much lower than that of I27 (∼230 pN) [1] indicating that M-crystallin is mechanically weaker compared to I27. On the other hand, the contour length change for M-crystallin is longer than that of I27 despite its smaller size (4aa less compared to I27). This is due to the longer N-C distance of I27 (∼4.3 nm) compared to ∼1.5 nm of M-crystallin in the native state.

**Table 2:** Mechanical properties of M-crystallin.

Sample	Contour length change (nm)†	Unfolding force (pN)*	Δx_u_ (nm)	k_u_^0^ (1/s)	ΔG‡ (kcal/mol)	k_s_ (N/m)
Apo	29.1±0.7	91±21	0.55±0.02‡	1.1×10^−2^–1.0×10^−4^ ‡ ^a^	16.4±1.4‡ ^b^	0.75¶
	(n = 254)		0.49±0.02§	0.07±0.03§		
Holo	28.9±0.6	125±20	0.38±0.02‡	1.1×10^−2^–5.0×10^−4^ ‡ ^a^	15.9±0.9‡ ^b^	1.52¶
	(n = 194)		0.34±0.02§	0.11±0.05§		

† The ‘n’ in the parentheses is the number of events used in the analysis.

*mean ± SD.

‡ Monte Carlo simulation.

§ Bell-Evans-Ritchie approximation.

a the range is obtained by using the average unfolding force ± SD in Monte Carlo simulations (see Supporting Material in File S1).

b the errors obtained from the range of k_u_^o^.

¶ k_s_ calculated from Monte Carlo values of k_u_^0^ and Δx_u._

### Ca^2+^ binding effect on the mechanical resistance of M-crystallin

**Figure 3:**
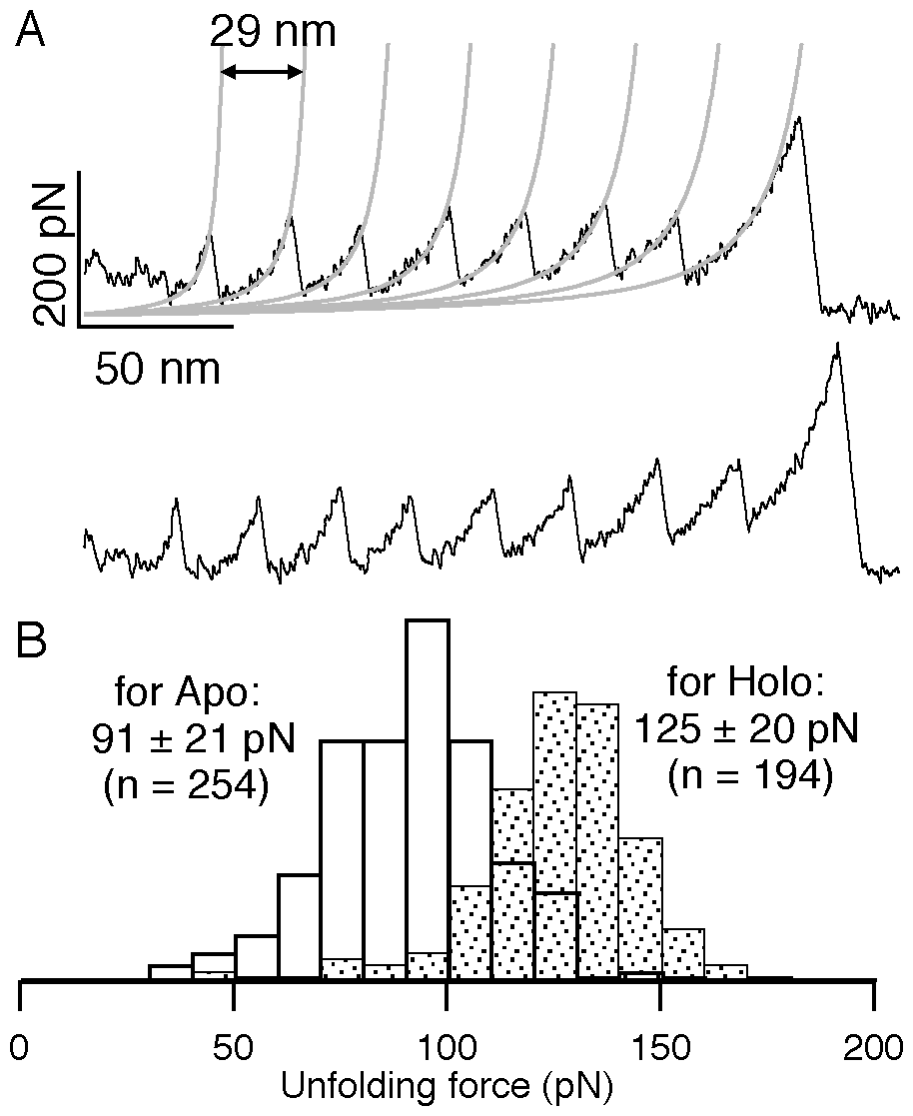
Mechanical unfolding of Ca^2+^ bound (M-crystallin)_8_. (*A*) A pair of FX traces obtained in the presence of 10 mM Ca^2+^. The contour length change upon unfolding is ∼29 nm and the unfolding force is ∼125 pN in the sawtooth curves (black). WLC fits are also shown (grey). (*B*) The unfolding force histograms of (M-crystallin)_8_ in holo (filled) and apo (unfilled) show that Ca^2+^ stabilizes the protein mechanically by ∼35 pN.

Next, we measured the effect of ligand binding on the mechanical unfolding by doing pulling experiments on M-crystallin octamer under ligand saturating conditions ([Ca^2+^] = 10 mM). At this condition, nearly all M-crystallin units in octamer are saturated with Ca^2+^ binding. A pair of representative unfolding FX traces of holo protein at a pulling speed of 1000 nm/s is shown in Figure 3. WLC fits measured a contour length change of ∼29 nm upon holo protein unfolding. This is exactly the same as that of apo protein. However, the unfolding forces of crystallin increased from 91 pN of apo protein to 125 pN upon Ca^2+^ addition. A histogram of unfolding forces is shown in Figure 3*B*. The measured unfolding force of holo protein is 125±20 pN at the same pulling speed (Table 2). The histogram from apo protein experiments (Figure 3*B*) is also shown, to directly compare it with holo protein. It is evident from data that there is a ∼35 pN shift between apo and holo histograms. We have also analyzed peak-wise unfolding forces of all peaks (1 to 8) (Supporting Material, Figure S5 in File S1). The difference in unfolding force between apo and holo protein is 30–35 pN at all peaks (1 to 8), which also confirms that the effect of Ca^2+^ is genuine.

Furthermore, a pulling experiment was performed on apo protein and measured its mechanical properties. Then Ca^2+^ was added to the apo protein and pulling was resumed on the Ca^2+^ bound holo protein using the same cantilever in order to rule out systematic errors, if any, caused because of using different cantilevers to do pulling experiments (Figure S6 in File S1). Indeed, the results from this experiment also confirmed that Ca^2+^ bound M-crystallin unfolds at higher forces than apo protein. Hence, from the pulling experiments on apo and holo proteins, we can clearly conclude that Ca^2+^ binding enhances the mechanical stability of M-crystallin.

**Figure 4:**
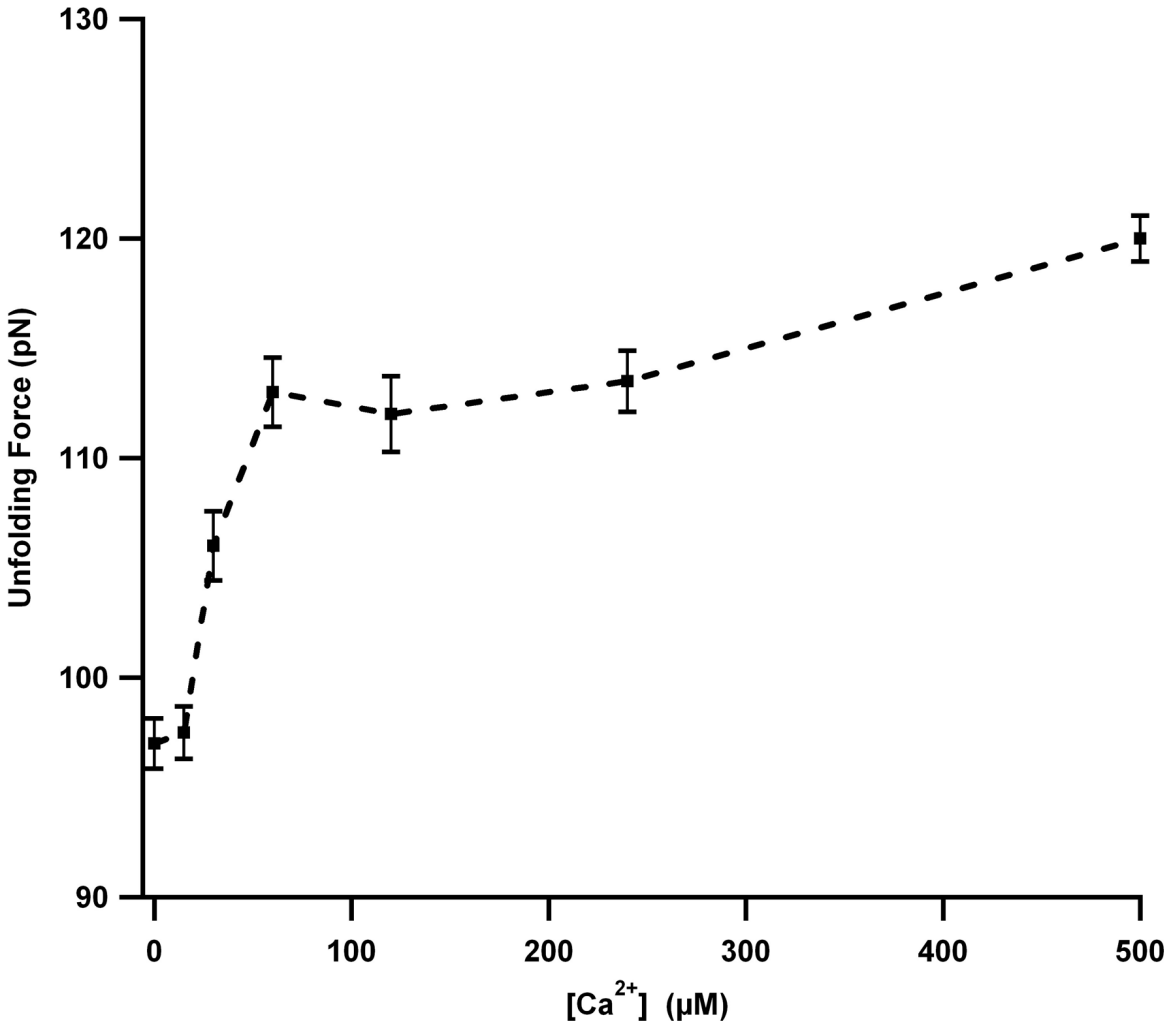
[Ca^2+^] dependent unfolding forces of (M-crystallin)_8_. The unfolding force histograms at various Ca^2+^ concentrations are shown in Figure S7 in File S1. The increase in unfolding force in two phases is consistent with two Ca^2+^ binding sites (see text for more details).

As shown in Figure 1, there are two Ca^2+^ binding sites (site I with K_d1_ ∼30 µM and site II with K_d2_∼200 µM) in M-crystallin with different binding strengths (Table 1). At [Ca^2+^]<100 µM, site I is preferred, and above this concentration, both sites will be bound. Hence, it would be possible to do pulling experiments on apo protein, protein with site I predominantly bound, and holo protein with both sites bound, by gradual titration with Ca^2+^ to find out if the mechanical stability of partially bound M-crystallin is different from that of apo and holo proteins. Therefore, we probed the effect of Ca^2+^ on the unfolding force of M-crystallin by doing pulling experiments with varying concentrations (10–500 µM) (Figure 4 and S7 in File S1). The graph clearly shows that the average of unfolding force distribution of M-crystallin increased upon adding Ca^2+^ in two phases; initially from ∼99 pN to ∼112 pN in 0–100 µM Ca^2+^ and then from 112 pN to 120 pN in 100–500 µM Ca^2+^. It must be noted that at the Ca^2+^ concentrations used in the titration experiment, there would be a mixture M-crystallin populations (apo, holo and that with one of the sites bound with Ca^2+^) is present in solution. Nevertheless, according to the ITC results where it was found that the dissociation constants for the two Ca^2+^ binding sites are ∼30 µM and ∼170 µM in the polyprotein (Figure S4 in File S1 and Table 1), it is very likely that the first phase in Figure 4 is due to the binding of Ca^2+^ predominantly at site I and the later transition at ∼200 µM is due to the binding of Ca^2+^ to the site II.

### Speed dependent mechanical stability and simulations

**Figure 5:**
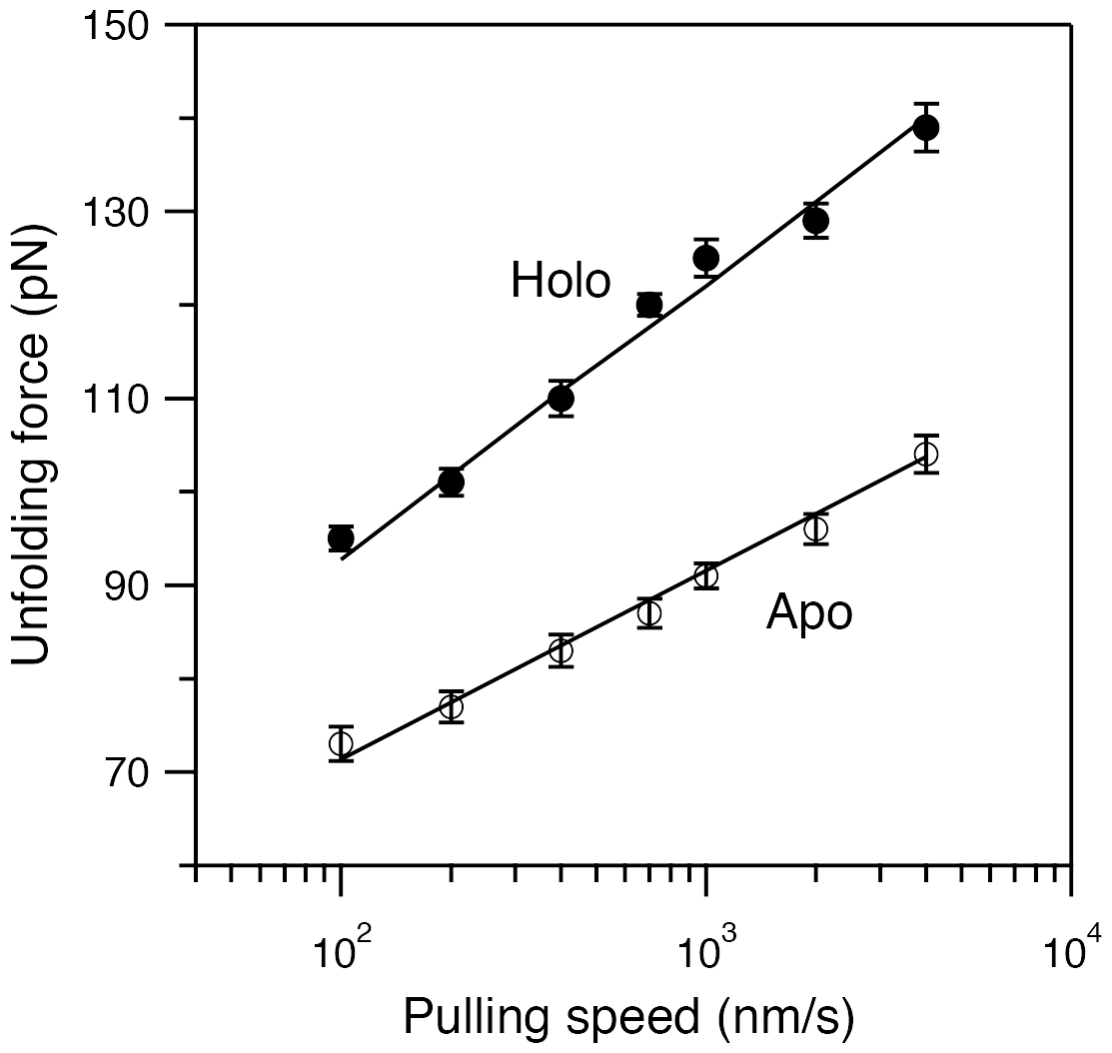
Pulling speed dependence on mechanical unfolding of (M-crystallin)_8_. A semi-logarithmic plot of unfolding force versus pulling speed for apo protein (○) and holo protein (•). Errors bars in the experimental data are SE. The Monte Carlo fits (solid line) are also shown for apo and holo proteins. Results from Monte Carlo simulations are given in Table 2. It is evident from the data that Ca^2+^ binding mechanically stabilizes M-crystallin by ∼30 pN at all pulling speeds.

**Figure 6:**
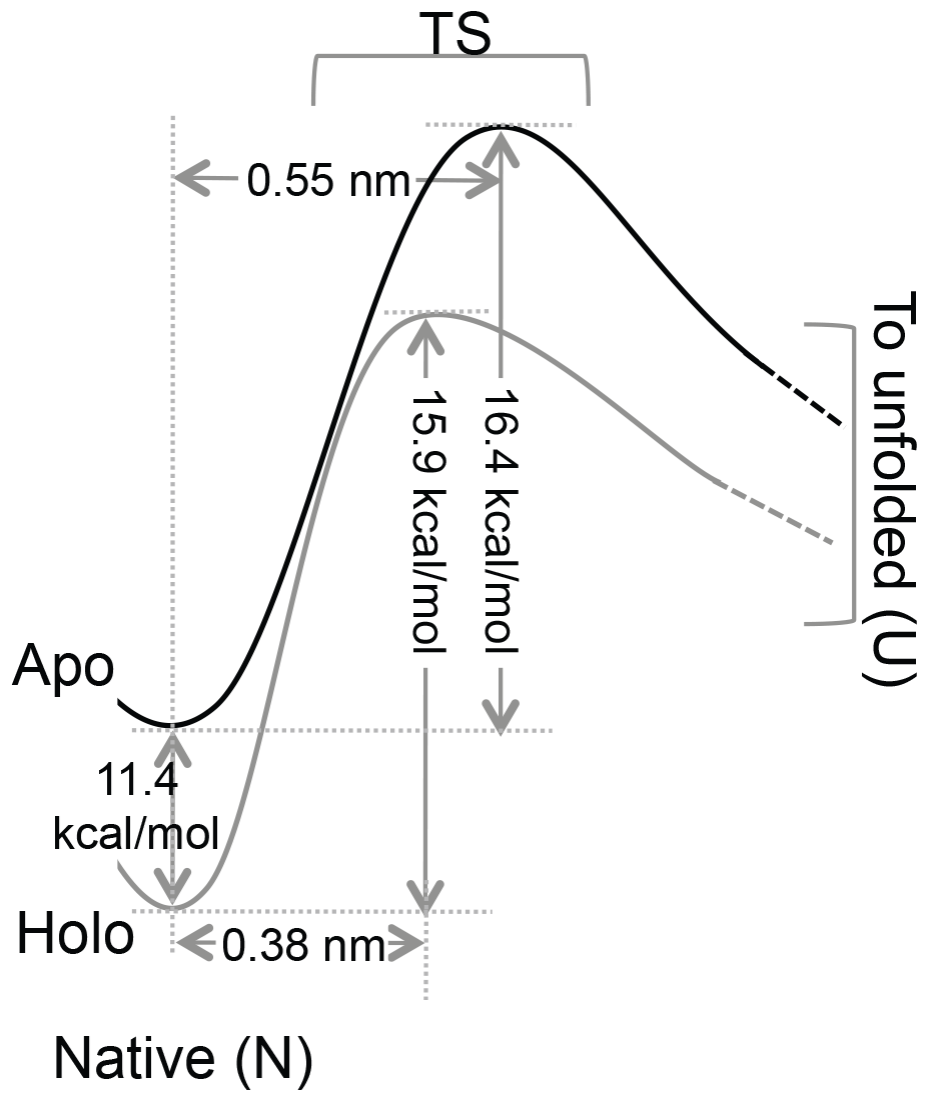
A schematic of two-state energy level diagram depicting the thermodynamic and mechanical stabilization of M-crystallin upon Ca^2+^ binding. Ligand binding not only stabilizes the native state (N) by ΔG∼11.4 kcal/mol but also reduces the unfolding potential width (Δx_u_) from 0.55 nm to 0.38 nm. Estimates of unfolding transition state (TS) energy barriers (ΔG^‡^) are also indicated. See text and Tables 1 and 2 for more details.

To further investigate the Ca^2+^ enhanced mechanical stability, we have measured the speed dependent mechanical unfolding parameters by varying the pulling speed in the range 100–4000 nm/s. A semi-logarithmic plot of the unfolding force versus the pulling speed is shown in Figure 5 for apo and holo proteins. It is evident that the unfolding forces of apo protein are always lower than that of the Ca^2+^ bound M-crystallin in the entire pulling speed range. We have further performed Monte Carlo simulations [1], [27] assuming a simple two-state energy landscape with a single energy barrier to reproduce the experimental data shown in Figure 5. Fits by the Monte Carlo simulations are shown in Figure 5 and results are presented in Table 2. In the two-state model, the spontaneous unfolding rate k_u_^0^∼1.1×10^−2^–1.0×10^−4^ s^-1^ and the distance to the transition state Δx_u_ = 0.55±0.02 nm have fitted well to the experimental data of apo protein (Figure S8 and Table S1 in File S1). The k_u_^0^ value of the holo protein 1.1×10^−2^–5.0×10^−4^ s^−1^, is in the same order of magnitude as that of the apo protein. However, there is a significant decrease in the Δx_u_ value, from 0.55±0.02 nm for apo to 0.38±0.02 nm for holo, upon Ca^2+^ binding. The results from Monte Carlo simulations are depicted by an energy landscape (Figure 6). To confirm that the decrease in Δx_u_ upon Ca^2+^ binding is genuine, we have also used Bell-Evans-Ritchie approximation as described previously [29]–[31], [34] to fit the pulling speed dependent data. Using this model, we obtained Δx_u_∼0.49±0.02 nm for apo and 0.34±0.02 for holo. This reconfirms that the decrease in Δx_u_ of M-crystallin upon Ca^2+^ binding is genuine and significant. Also, the k_u_^0^ values are in the same order of magnitude between apo and holo, which is also the case in Monte Carlo simulations. The results are given in Table 2.

## Discussion

It is important to show experimentally that polyprotein construction of M-crystallin does not modify its structural and Ca^2+^ binding properties in the octamer. The structural properties of M-crystallin are unchanged upon construction of its octamer as shown by CD and fluorescence spectroscopy. CD reports the secondary structure in the far-UV region and the tertiary structure in the near-UV region. In our observations, the data for octamer is indistinguishable from that of the monomer and this is an indication that the structure is not altered in polyprotein construction. Recently, we have shown by NMR that M-crystallin dimer has identical structural properties as that of the monomer [24]. Furthermore, Ca^2+^ binding was probed using CD, fluorescence and ITC and the measured properties are similar between monomer and octamer. The data obtained from ITC experiments shows that there are two Ca^2+^ binding sites with different dissociation constant (K_d_) values. The extracted K_d_ values from these experiments (31 µM and 166 µM) are similar to that of the monomer. All these results indicate that the structure and Ca^2+^ binding property are unperturbed on making M-crystallin octamer using polyprotein engineering.

### Comparison of mechanical properties of M-crystallin with other β-sandwich proteins

Mechanical stability of M-crystallin, for both apo and holo conditions, is lower than that of I27 in the pulling speed range 100–4000 nm/s. However, it is not as labile as α-helical proteins (e.g., spectrin), which unfolds below ∼30 pN at 300 nm/s [35]. Hence, we can say that β-sandwich topology with Greek Keys (Figure 1) of M-crystallin makes it mechanically resistant, but lack of “mechanical clamp” in its structure makes it differ from I27 in its mechanical properties. The observed contour length change of ∼29 nm confirms that M-crystallin unfolds in all-or-none manner without any discernible intermediates during mechanical unfolding. From its structure, it can be seen that the termini in the protein (β-strands A and H; shown in Figure 1, *C* and *D*) are in twoβ-sheets facing each other. Mechanical stretching along the line joining N,C-termini makes these strands unravel in a “peeling geometry” as opposed to “shearing geometry” of the “mechanical clamp” in I27. And this could be a plausible reason for the lower unfolding force of M-crystallin compared to that of I27, despite having a β-sandwich topology. Previously, as reported for a yellow fluorescent protein (EYFP) by Perez-Jimenez *et al* [36], the “shearing geometry” requires much higher mechanical force than the “peeling geometry”. In the β-barrel protein EYFP, the main unfolding peak where the unfolding occurs in a “peeling geometry” requires a force of ∼60 pN whereas the intermediate unfolds through “shearing geometry” at ∼120 pN. In the case of C2 protein of human synaptogamin 1, its domains C2A and C2B with β-sandwich topology were shown to unfold at 50 and 100 pN, respectively [37]. In C2 domain structure, the peripheral strands are anti-parallel to each other and also held together by backbone H-bonding. In this geometry, the bridging H-bonds are parallel to the N-C pulling axis and hence they break in a “zipper-like” fashion causing the protein to unfold at a lower force. So, this study is in concurrence with earlier reports that the peeling and unzipping geometries of unraveling are mechanically less resistant than unfolding by shearing.

### Ca^2+^ binding effects on protein mechanical properties

The unfolding force of M-crystallin is increased by ∼35 pN upon Ca^2+^ binding. From the equilibrium chemical denaturation experiments, it was reported that Ca^2+^ binding stabilizes M-crystallin such that the free energy for unfolding ΔG_U_–_N_ increases by 0.9 kcal/mol [16]. Previous studies showed the protein mechanical properties can be affected by small ligands [25], [38]–[41], ions [42]–[44] and large molecules [45], [46]. Earlier studies on Ca^2+^ binding effect on protein mechanical unfolding studies showed two kinds of behavior. In the first kind, the Ca^2+^ binding has mostly no effect on the mechanical stability of proteins as reported for calmodulin [18], [47], [48] and recoverin [49]. However, the Ca^2+^ binding effect is seen in the enhancement of the refolding kinetics of calmodulin and von Willebrand factor (vWF) A2 domain [47], [48], [50]. In the second kind, the Ca^2+^ binding has directly affected the mechanical stability of the protein as demonstrated in the case of C-cadherin ectodomain [44], vWF [51] and cohesion-dockerin complex [52]. As described by Oroz *et al* [44], the structure of C-cadherin ectodomain rigidifies upon Ca^2+^ binding and “canalizes” its nanomechanical behavior by generating a novel Ca^2+^ dependent mechanical element. Although, it seems that M-crystallin belongs to the second kind, it must be noted that there are differences in the Ca^2+^ binding sites between M-crystallin and C-cadherin ectodomain. In the case of cadherin the Ca^2+^ binding site is located at an inter-domain junction whereas in M-crystallin both Ca^2+^ binding sites are part of the same domain.

Furthermore, Ca^2+^ titration experiment showed that the mechanical stabilization of M-crystallin occurs in two phases, which is in concurrence with two Ca^2+^ binding sites with different binding constants. Interestingly, the increase in the unfolding force is similar in both phases (99–112 pN in 0–100 µM Ca^2+^ and 112–120 pN in 100–500 µM Ca^2+^) indicating that both Ca^2+^ binding sites confer mechanical stabilization in spite of different binding constants.

### Ca^2+^ binding reduces the unfolding potential width of M-crystallin

Another important aspect is the distance to the unfolding transition state (Δx_u_) extracted from experiments and simulations. The measured Δx_u_ for apo M-crystallin is 0.55 nm, which is slightly longer than that of immunoglobulin-like domains I1 (0.35 nm) and I27 (0.25 nm) from muscle protein titin [1], [3]. The Δx_u_ is the magnitude of deformation along the pulling direction that occurs in protein to reach the transition state before crossing the unfolding energy barrier and it is usually taken as a measure of protein deformation response [32]. From our experimental results we can say that M-crystallin is flexible and needs to deform more in magnitude compared to I27 in reaching the unfolding transition state from its native structure. Also, it is not that rare to observe a large values for Δx_u_, which is the case for GFP where pulling in different directions resulted in a wide range (0.12–0.45 nm) of Δx_u_ values [32]. Interestingly, the Δx_u_ of M-crystallin becomes smaller (0.38 nm) upon Ca^2+^ binding. To independently estimate Δx_u_ and k_u_^0^, we have used Bell-Evans-Ritchie approximation as described previously [28]–[30]. The estimates from this model are also given in Table 2. The Δx_u_ values from this model are in excellent agreement with that of Monte Carlo simulations. The k_u_^0^ values from this model differ from Monte Carlo simulations by about an order of magnitude. Both Monte Carlo simulations and Bell-like model give more accurate estimates of Δx_u_ than k_u_^0^. It is possible that Ca^2+^ binding makes the protein more brittle, which then requires a minimal deformation but higher force to reach the unfolding transition state. In other words, the transition state Ca^2+^ bound M-crystallin might have a different structure and it is possible that a different set of interactions might be broken along the unfolding reaction coordinate which also explains the distance to the transition state. Furthermore, we have calculated the deformation spring constant of the protein (k_s_) assuming a simple harmonic unfolding potential (see Materials and Methods section). The estimated k_s_ for apo protein is 0.75 N/m and that of holo protein is 1.52 N/m, which suggests that Ca^2+^ bound M-crystallin is twice as stiff as apo protein. In fact, NMR investigations on apo and holo structures revealed that the Ca^2+^ bound M-crystallin has more ordered structure than apo protein [16], suggesting that the holo protein is much more brittle and less flexible than the apo form. Thus, the effect of Ca^2+^ binding on the mechanical properties M-crystallin is to reduce the width of the unfolding potential well (Figure 6).

## Conclusion

M-crystallin octamer has been constructed using polyprotein engineering and its structural and Ca^2+^ binding properties studied by using fluorescence, CD and ITC techniques are shown to be unaffected. Furthermore, SMFS studies on octamer showed that M-crystallin unfolds in a two-state manner, albeit at a lower unfolding force (91 pN) than that of I27 at a pulling speed of 1000 nm/sec. Despite having β-sandwich structure, I27 and M-crystallin have different mechanical stabilities and this has been attributed to their topology near the termini, which makes I27 to unfold via “shearing geometry” whereas M-crystallin unravels through “peeling geometry”. Moreover, Ca^2+^ binding increases the mechanical unfolding force of M-crystallin to 125 pN by reducing the width of its unfolding potential from 0.55 nm to 0.38 nm. These results demonstrate that Ca^2+^ as a ligand can also affect the mechanical properties of proteins by altering the unfolding energy landscape, in addition to previously reported mechanisms where it selectively influences the refolding pathways of proteins [48], [50].

## Supporting Information

File S1 SDS PAGE analysis and Size Exclusion chromatograms; CD spectra; Steady state fluorescence spectra; ITC experimental data; SMFS experimental data obtained using a single cantilever; Peak-wise unfolding forces; Unfolding force histograms from Ca^2+^ titration experiment; Monte Carlo simulation fits; Table showing the unfolding parameters obtained from Monte Carlo simulations. **Table S1.** Range of the unfolding rate (k_u_^0^) and the distance to the unfolding transition state Δx_u_ by fitting the unfolding force (average), average-SD, and average+SD to Monte Carlo (MC) simulations. **Figure S1.** Gel electrophoresis results of purified monomer and octamer of M-crystallin. SDS PAGE of M-crystallin monomer (*A*) and octamer (*B*) showing bands at ∼11 kDa and ∼90 kDa, respectively. Size exclusion chromatogram of M-crystallin eluted at Superdex 75 and 200 columns for monomer (*C*) and octamer (*D*) respectively indicating their high purity level in native conditions (10 mM Tris buffer with 50 mM KCl, pH 7.5). **Figure S2.** Circular dichroism (CD) spectra of monomer and octamer of M-crystallin. Far-UV CD spectra of M-crystallin monomer (*A*) and octamer (*B*). Near-UV CD spectra of M-crystallin monomer (*C*) and octamer (*D*). Apo protein spectra are shown in black color and holo protein spectra in grey color. **Figure S3.** Steady state fluorescence spectra of monomer and octamer of M-crystallin. Emission spectra of M-crystallin apoform monomer (black solid line) and octamer (grey solid line) in native conditions. Emission spectra in denaturing condition (6 M GdnHCl) for monomer (black dashed line) and octamer (grey dashed line). The spectra of holoform were identical. **Figure S4.** Ca^2+^ binding measurements using isothermal titration calorimetry (ITC) for M-crystallin monomer (*A*) and octamer (*B*). (Top) Reaction heats measured from stepwise calorimetry performed with 5 mM CaCl_2_ injected against 180 µM M-crystallin in the cell. (Bottom) Binding isotherms are fitted with two-site sequential binding model and results are given in Table 1. **Figure S5.** Peak-wise unfolding forces of apo and holo protein of M-crystallin at the pulling speed of 1000 nm/sec. There is 30–35 pN enhancement in the mechanical stability of M-crystallin upon Ca^2+^ binding. The errors are standard deviations. **Figure S6.** The unfolding force histograms from the pulling experiment done on apo and holo (M-crystallin)_8_ using the same cantilever, also concur with Fig. 3(*B*). **Figure S7.** Histograms of unfolding forces of M-crystallin at different Ca^2+^ concentrations. The errors indicate standard deviation. Histogram of 30 µM Ca^2+^ data is plotted with smaller bin-size to show that bimodal distribution is not observed. **Figure S8.** Monte Carlo simulation fits to speed dependent unfolding forces. Unfolding forces (average), average-SD, and average+SD were separately fitted using Monte Carlo simulations to extract the range of k_u_^o^ and Δx_u_. Results are given in Table S1.
(DOCX)
