# Correction: Ca^2+^ Binding Enhanced Mechanical Stability of an Archaeal Crystallin

**DOI:** 10.1371/journal.pone.0102924

**Published:** 2014-07-11

**Authors:** The PLOS ONE Staff

Figure 1 is incorrectly printed in black and white. Please see the correct Figure 1 here.

**Figure 1:**
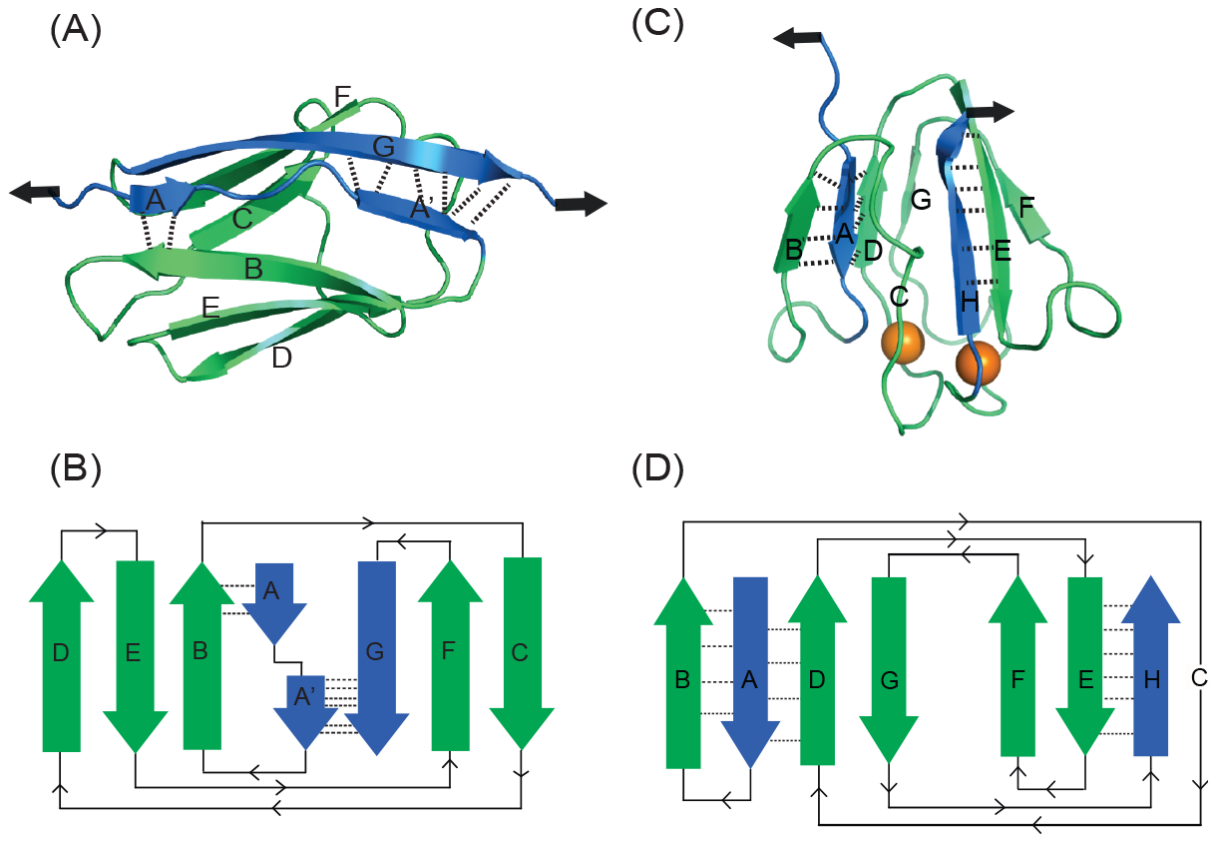
Structure and 2D topology diagram of two β-sandwich proteins with Greek key motifs used in this study. The pulling direction used in the single-molecule force spectroscopy (SMFS) experiments is shown by arrows. (*A*) NMR structure of I27 (PDB ID: 1TIT). Terminal β-strands A′ and G are directly connected by H-bonding, shearing this “mechanical-clamp” results in the mechanical unfolding of the protein. The rupture of H-bonds between A and B strands constitutes the less stable mechanical intermediate. (*B*) 2D topology diagram of I27. The five-stranded (BCDEF) ‘double’ Greek key (3,2)_3_ formed by overlapping (3,1)_N_ and (2,2)_C_ Greek keys (as defined by Hutchinson and Thornton [53]). (*C*) NMR structure of M-crystallin (PDB ID: 2K1W) bound to two Ca^2+^ ions (shown as black spheres). The terminal β-strands A and H are not directly bonded and they need to be “peeled” away from each other to unfold the protein. (*D*) 2D topology diagram of M-crystallin showing the two (3,1)_C_ Greek keys formed by ABCD and EFGH. In both cases, the backbone H-bonding around the terminal strands is shown.
