# Supplementary material for: Ca2+ Binding Enhanced Mechanical Stability of an Archaeal Crystallin
Source: PLoS One. 2014 Apr 11;9(4):e94513. doi: 10.1371/journal.pone.0094513 (PMC3984160; doi:10.1371/journal.pone.0094513)
Supplement: File S1 — SDS PAGE analysis and Size Exclusion chromatograms; CD spectra; Steady state fluorescence spectra; ITC experimental data; SMFS experimental data obtained using a single cantilever; Peak-wise unfolding forces; Unfolding force histograms from Ca2+ titration experiment; Monte Carlo simulation fits; Table showing the unfolding parameters obtained from Monte Carlo simulations. Table S1. Range of the unfolding rate (ku 0) and the distance to the unfolding transition state Δxu by fitting the unfolding force (average), average-SD, and average+SD to Monte Carlo (MC) simulations. Figure S1. Gel electrophoresis results of purified monomer and octamer of M-crystallin. SDS PAGE of M-crystallin monomer (A) and octamer (B) showing bands at ∼11 kDa and ∼90 kDa, respectively. Size exclusion chromatogram of M-crystallin eluted at Superdex 75 and 200 columns for monomer (C) and octamer (D) respectively indicating their high purity level in native conditions (10 mM Tris buffer with 50 mM KCl, pH 7.5). Figure S2. Circular dichroism (CD) spectra of monomer and octamer of M-crystallin. Far-UV CD spectra of M-crystallin monomer (A) and octamer (B). Near-UV CD spectra of M-crystallin monomer (C) and octamer (D). Apo protein spectra are shown in black color and holo protein spectra in grey color. Figure S3. Steady state fluorescence spectra of monomer and octamer of M-crystallin. Emission spectra of M-crystallin apoform monomer (black solid line) and octamer (grey solid line) in native conditions. Emission spectra in denaturing condition (6 M GdnHCl) for monomer (black dashed line) and octamer (grey dashed line). The spectra of holoform were identical. Figure S4. Ca2+ binding measurements using isothermal titration calorimetry (ITC) for M-crystallin monomer (A) and octamer (B). (Top) Reaction heats measured from stepwise calorimetry performed with 5 mM CaCl2 injected against 180 µM M-crystallin in the cell. (Bottom) Binding isotherms are fitted with two-site sequential binding model and resu [file pone.0094513.s001.docx]

Supporting Information

**Ca^2+^ binding enhanced mechanical stability of an archaeal crystallin**

Venkatraman Ramanujam, Hema Chandra Kotamarthi and Sri Rama Koti Ainavarapu*

*Department of Chemical Sciences, Tata Institute of Fundamental Research, Homi Bhabha Road, Colaba, Mumbai 400005, India*

*Correspondence: koti@tifr.res.in

**Circular dichroism (CD)**

The CD spectra were recorded on a JASCO J-715 spectropolarimeter. A spectral range of 205−250 nm in far-UV region was used for probing the secondary structure of the protein and a spectral range of 250−300 nm in the near-UV region was used for probing the tertiary structure of the protein. Far-UV CD spectra were collected on 2 µM octamer and on 15 µM monomer M-crystallin in 10 mM Tris buffer (pH 7.5) containing 50 mM KCl for apo condition and 10 mM Tris buffer (pH 7.5) containing 50 mM KCl, 10 mM CaCl_2_ for holo condition with 1 mm path length cuvette. Near-UV CD spectra were collected on 80 µM monomer and 10 µM of octamer protein in 10 mM Tris buffer (pH 7.5) containing 50 mM KCl and 10 mM CaCl_2_ using 1 cm path length cuvette. All CD spectra were acquired at a scan speed of 50 nm/min and with a response time of 2 s.

**Fluorescence spectroscopy**

Fluorescence spectra were acquired on a spex Fluoromax-3 spectrofluorometer. Spectra were acquired for M-crystallin octamer and monomer at 1 µM and 10 µM, respectively, in 10 mM Tris buffer (pH 7.5) containing 50 mM KCl for apo protein and 10 mM Tris buffer (pH 7.5) containing 50 mM KCl and 10 mM CaCl_2_ for holo protein. The path length of the cuvette was 1 cm. The protein was excited at 295 nm and the fluorescence emission was collected from 310 to 400 nm. The excitation and emission bandwidths were 2 nm. Each spectrum was collected with a 1 s integration time.

**Isothermal titration calorimetry (ITC)**

ITC experiments were carried out on a VP isothermal titration calorimeter from Microcal, Inc (Northampton, MA). Samples were centrifuged and degassed before titration. Protein (180 µM) and CaCl_2_ (5 mM) in 10 mM Tris buffer (pH 7.5) and 50 mM KCl were used for titrations. The titration was performed at 298 K. Stirring speed was set at 300 rpm, and after baseline stabilization 25 injections of 10 µl volume were carried out within an interval of 20 s. The dilutions of ligand into buffer and buffer into protein were carried out, and these dilutions were incorporated before data analysis. The data were analyzed using ORIGIN 7.0 (supplied with Microcalorimeter). The amount of heat released per addition of titrant fitted best to two-site sequential binding model [1].

TABLE S1: Range of the unfolding rate (k_u_^0^) and the distance to the unfolding transition state Δx_u_ by fitting the unfolding force (average), average-SD, and average+SD to Monte Carlo (MC) simulations.

| Sample | Unfolding force used for MC | Δx_u_  (nm) | k_u_^0^  (1/s) | ΔG^#^ (kcal/mol) |
| --- | --- | --- | --- | --- |
|  | Average-SD | 0.53 | 1.0 x 10^-2^ | -15.0 |
| Apo | Average | 0.55 | 0.9 x 10^-3^ | -16.4 |
|  | Average+SD | 0.57 | 1.0 x 10^-4^ | -17.8 |
|  |  |  |  |  |
|  | Average-SD | 0.36 | 1.1 x 10^-2^ | -15.0 |
| Holo | Average | 0.38 | 2.4 x 10^-3^ | -15.9 |
|  | Average+SD | 0.40 | 5.0 x 10^-4^ | -16.8 |

FIGURE S1


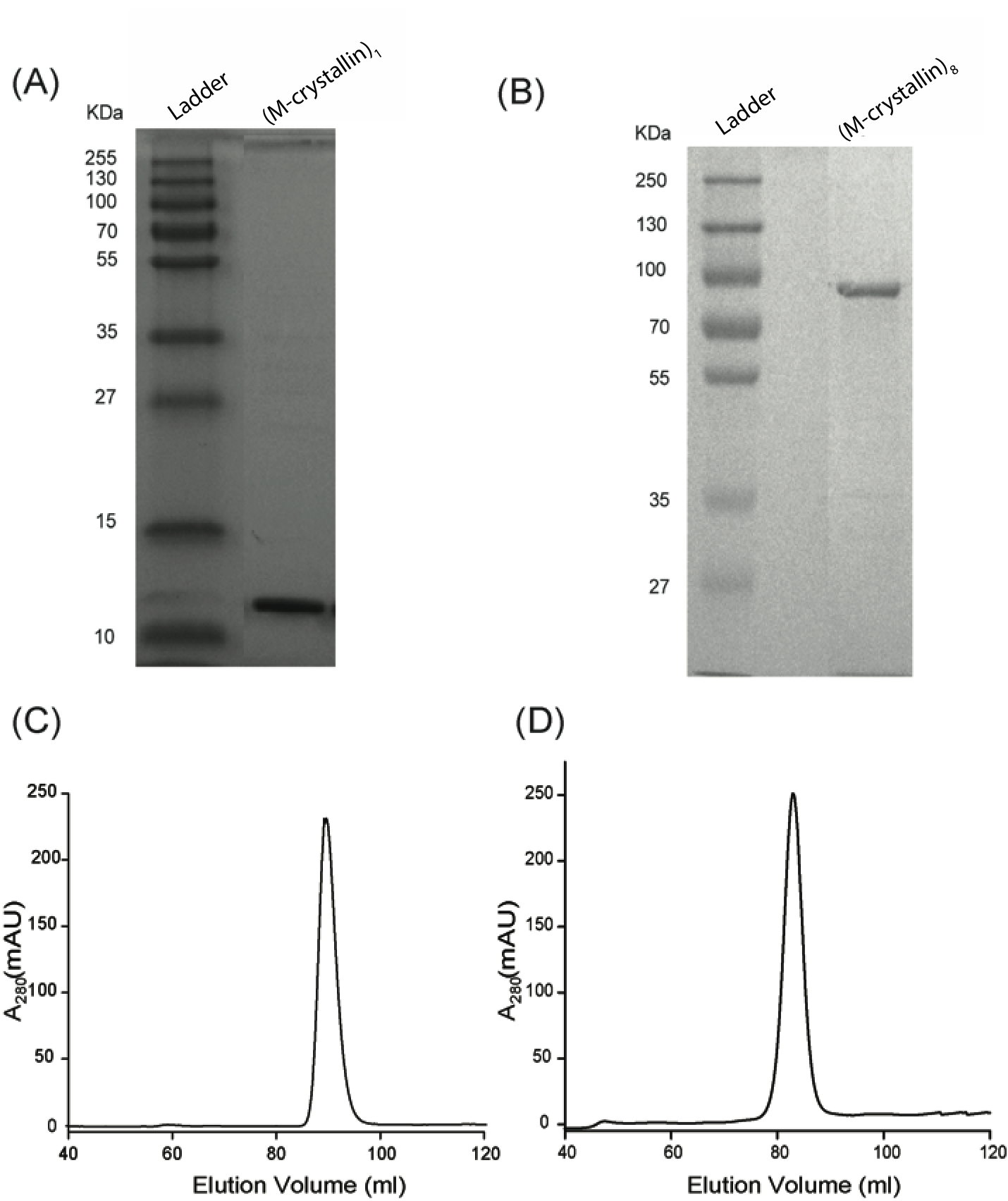


FIGURE S1: Gel electrophoresis results of purified monomer and octamer of M-crystallin. SDS PAGE of M-crystallin monomer (*A*) and octamer (*B*) showing bands at ~11 kDa and ~90 kDa, respectively. Size exclusion chromatogram of M-crystallin eluted at Superdex 75 and 200 columns for monomer (*C*) and octamer (*D*) respectively indicating their high purity level in native conditions (10 mM Tris buffer with 50 mM KCl, pH 7.5).

FIGURE S2


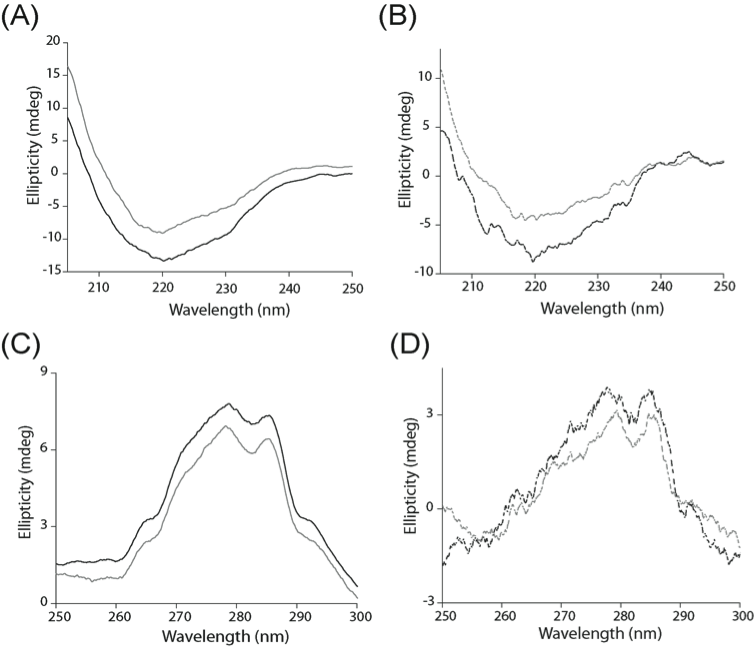


FIGURE S2: Circular dichroism (CD) spectra of monomer and octamer of M-crystallin. Far-UV CD spectra of M-crystallin monomer (*A*) and octamer (*B*). Near-UV CD spectra of M-crystallin monomer (*C*) and octamer (*D*). Apo protein spectra are shown in black color and holo protein spectra in grey color.

FIGURE S3


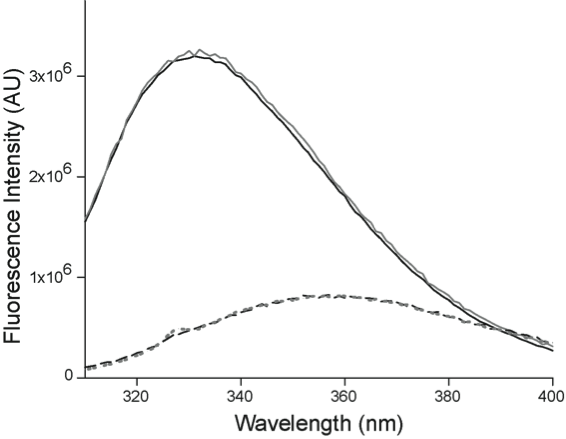


FIGURE S3: Steady state fluorescence spectra. Excitation wavelength is 295 nm. Emission spectra of M-crystallin apoform monomer (black solid line) and octamer (grey solid line) in native conditions. Emission spectra in denaturing condition (6M GdnHCl) for monomer (black dashed line) and octamer (grey dashed line). The spectra of holoform were identical.

FIGURE S4


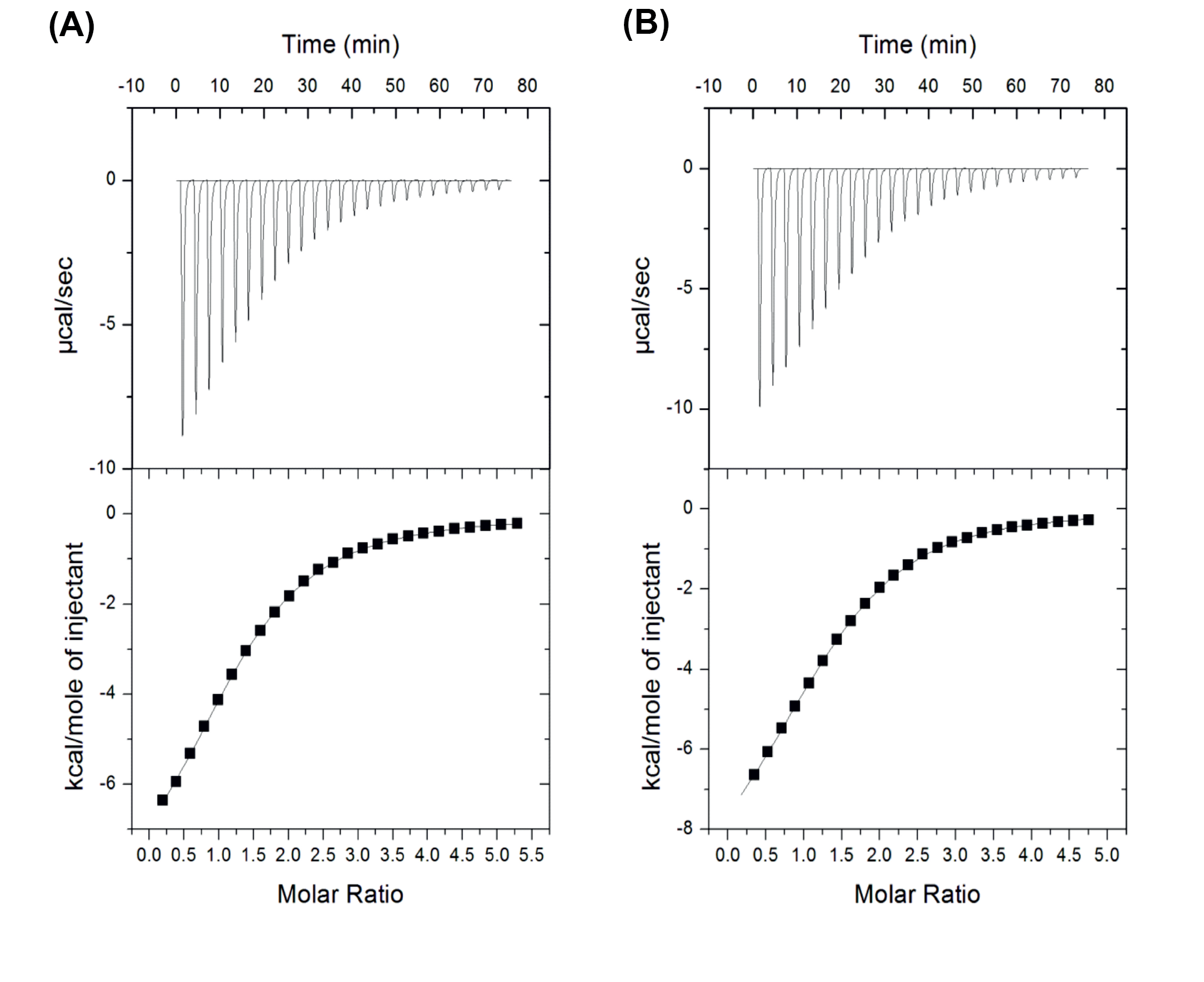


FIGURE S4: Ca^2+^ binding measurements using isothermal titration calorimetry (ITC) for M-crystallin monomer (*A*) and octamer (*B*). (Top) Reaction heats measured from stepwise calorimetry performed with 5 mM CaCl_2_ injected against 180 μM M-crystallin in the cell. (Bottom) Binding isotherms are fitted with two-site sequential binding model and results are given in Table 1.

FIGURE S5


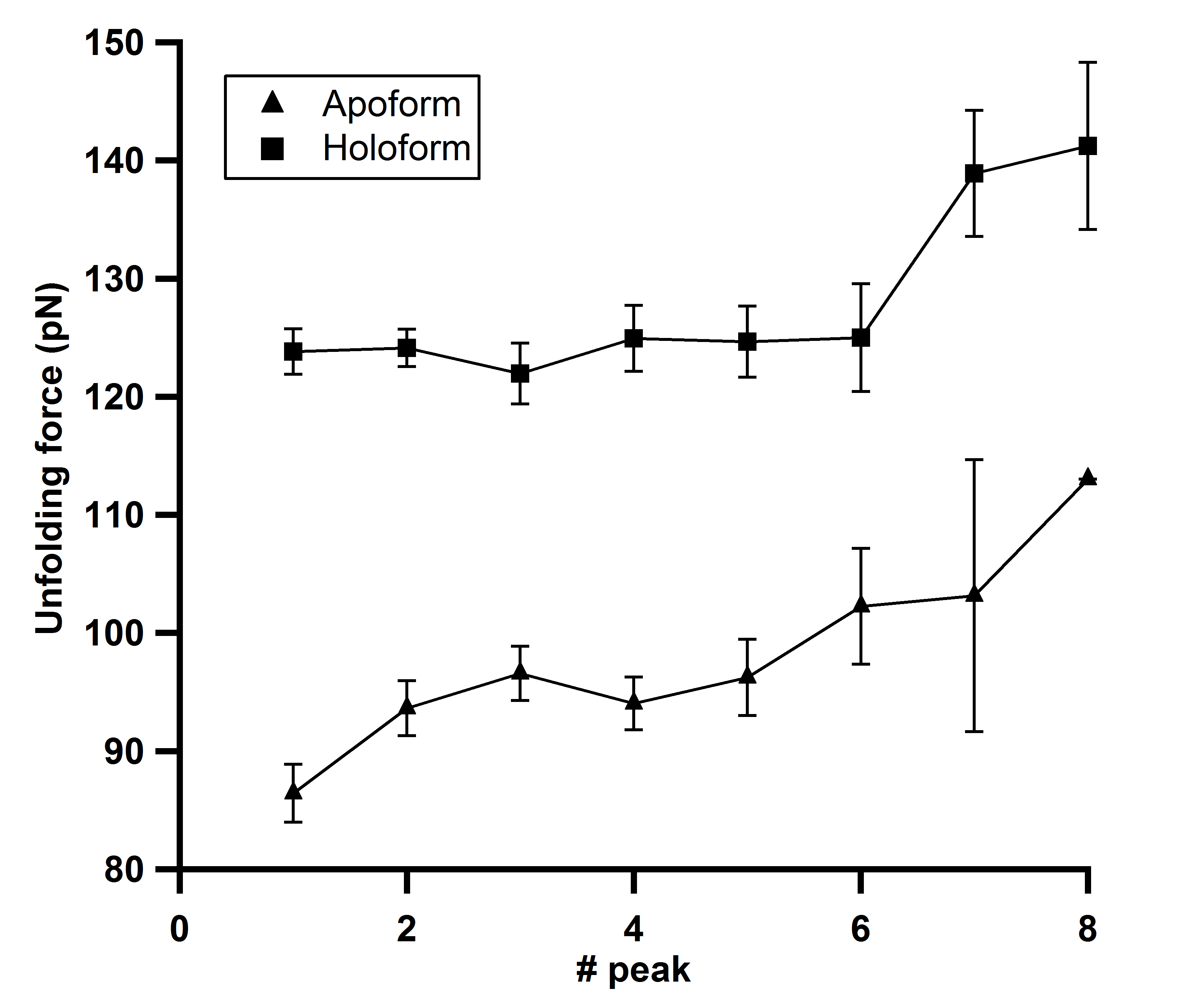


FIGURE S5: Peak-wise unfolding forces of apo and holo protein of M-crystallin at the pulling speed of 1000 nm/sec. There is 30-35 pN enhancement in the mechanical stability of M-crystallin upon Ca^2+^ binding. The errors are standard deviations.

FIGURE S6


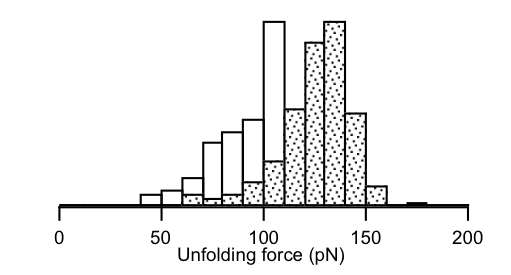


for Holo:

124 ± 17 pN (n= 162)

for Apo:

98 ± 20 pN (n= 111)

FIGURE S6: The unfolding force histograms from the pulling experiment done on apo and holo (M-crystallin)_8_ using the same cantilever, also concur with Fig. 3(*B*).

FIGURE S7


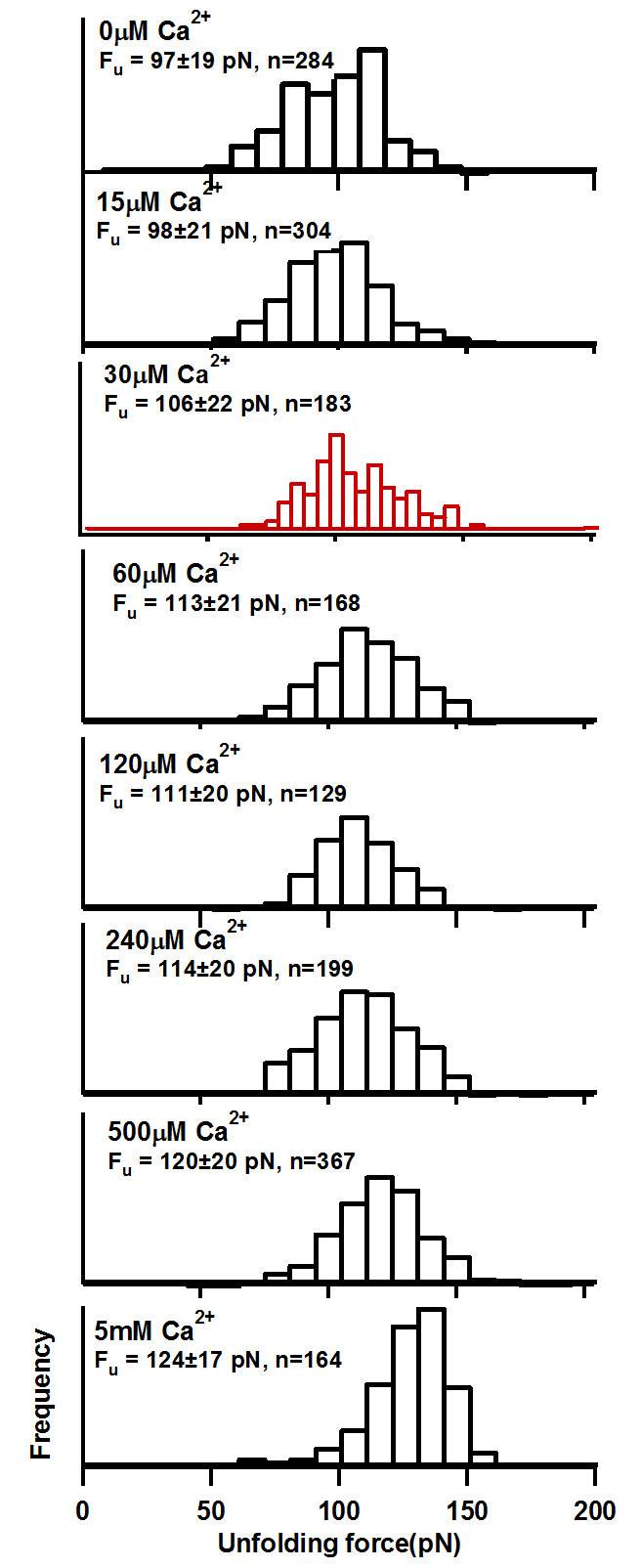


FIGURE S7: Histograms of unfolding forces of M-crystallin at different Ca^2+^ concentrations. The errors indicate standard deviation. Histogram of 30 μM Ca^2+^ data is plotted with smaller bin-size to show that bimodal distribution is not observed.

FIGURE S8


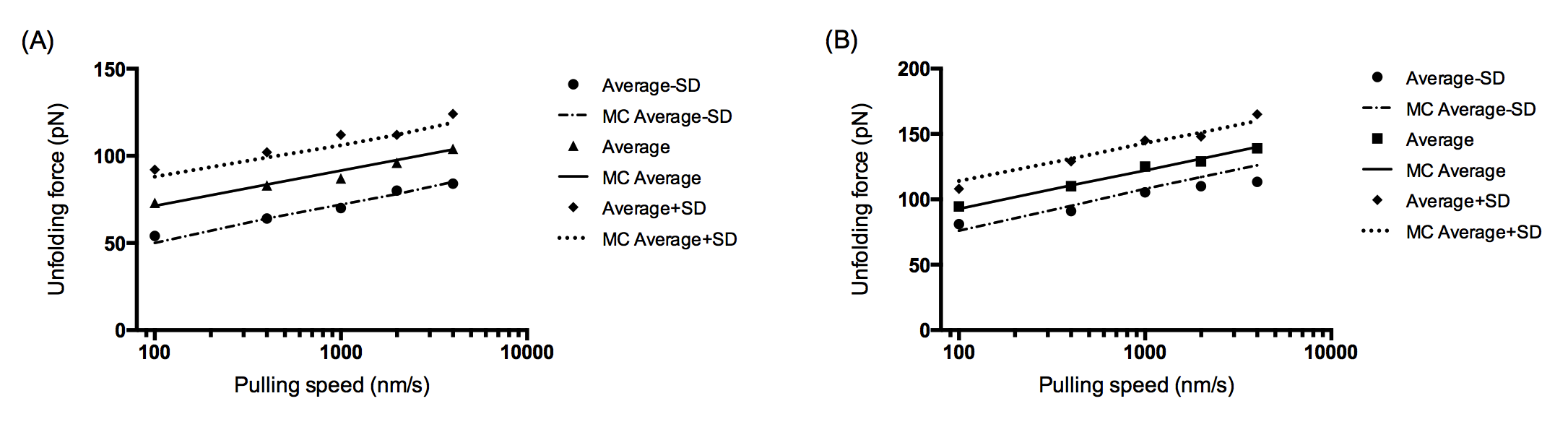


FIGURE S8: Monte Carlo simulation fits to speed dependent unfolding forces. Unfolding forces (average), average-SD, and average+SD were separately fitted using Monte Carlo simulations to extract the range of k_u_^o^ and Δx_u_. Results are given in Table S1.

**References**

1. Wiseman T, Williston S, Brandts J F, and Lin L N (1989) Rapid measurement of binding constants and heats of binding using a new titration calorimeter. Anal Biochem 179:131-137
